# Errata

**Published:** 2013-04-05

## Vol. 62, Suppl 1

In the *MMWR* supplement, “Advisory Committee on Immunization Practices (ACIP) Recommended Immunization Schedules for Persons Aged 0 Through 18 Years and Adults Aged 19 Years and Older — United States, 2013,” on page 7, in the third bulleted item under footnote 13, the text should read, “For children aged **2 months** through 10 years with high-risk conditions, see below.” On page 8, under Additional Vaccine Information, in the fourth bulleted item, the last reference should read, “American Academy of Pediatrics. **Immunization in Special Clinical Circumstances**. In: Pickering LK, Baker CJ, Kimberlin DW, Long SS, eds. Red book: 2012 report of the Committee on Infectious Diseases. 29th ed. Elk Grove Village, IL: American Academy of Pediatrics.”

